# Supplementary material for: Genome-Wide Identification and Expression Analysis of the CAMTA Gene Family in Roses (Rosa chinensis Jacq.)
Source: Plants (Basel). 2024 Dec 29;14(1):70. doi: 10.3390/plants14010070 (PMC11723114; doi:10.3390/plants14010070)
Supplement: Supplementary file 1 [file plants-14-00070-s001.zip › plants-3338324-supplementary.pdf]

Table S1. Details of the 10 conserved motifs of rose CAMTA proteins

Motif	Width(aa)	Motif Sequence
Motif 1	50	CMFGEVEVPAEVIADGWJRCYTPPHVPGRVPFYVTCSNRLPCSEVREFEY
Motif 2	50	DGHRWRKKKDGKTVKEAHERLKVGNVDVLHCYYAHGEDNPNFQRRSYWML
Motif 3	50	GQGVJHLGAALGYDWAILPIITAGVSVNFRDVNGWTALHWAAYFGREKTV
Motif 4	50	WKGRKDFLKIRKRIVKIQAHVRGHQVRKNYRKIVWSVGILEKVILRWRRK
Motif 5	49	LIGLGAAPGAVTDPTSKYPGGKTPADLASEKGHKGIAGYLSEVALTSHL
Motif 6	50	EAKQRWLRPAEICEILQNYKKFQITPEPPNQPPSGSLFLFDRKILRYFRK
Motif 7	36	GPSLSQDQLFSIVDFSPNWAYENSETKVLIVGKFLK
Motif 8	9	HIVLVHYRE
Motif 9	31	LKDSLAAVRNAAQAAARIQAAFRAHSFRRKQ
Motif 10	18	RVQSMVRSPEAREQYHRL

Table S2. Summary of cis-acting elements of *RcCAMTA* genes

Element	Sequence	Description	Number
TGA-element	AACGAC	auxin-responsive element	5
LTR	CCGAAA	cis-acting element involved in low-temperature responsiveness	6
TCA-element	CCATCTTTTT	cis-acting element involved in salicylic acid responsiveness	10
ABRE	ACGTG	cis-acting element involved in the abscisic acid responsiveness	21
ARE	AAACCA	cis-acting regulatory element essential for the anaerobic induction	13
G-box	CACGTG	cis-acting regulatory element involved in light responsiveness	12
CGTCA-motif	CGTCA	cis-acting regulatory element involved in the MeJA-responsiveness	7
O2-site	GATGATGTGG	cis-acting regulatory element involved in zein metabolism regulation	1
GC-motif	CCCCCG	enhancer-like element involved in anoxic specific inducibility	1
MBS	CAACTG	MYB binding site involved in drought-inducibility	5
GATA-motif	GATAGGA	part of a light responsive element	6
P-box	CCTTTTG	gibberellin-responsive element	5

Table S3. The sequences of primers used for qRT-PCR.

Gene Name	Forward Primer Sequence (5′-3′)	Reverse Primer Sequence (5′-3′)	E（%）	R^2
RcCAMTA1	GGTTGTAGCAGACGGTGTTCTTC	TCTTGAATTTGGTTGACTCGGTACTC	95.3	0.996
RcCAMTA2	AGAGGCTCAAGGCTGGAAGTG	CGGTAGTGGACGAGAACAATGTG	94.9	0.987
RcCAMTA3	TGGATGTTGACACTATTATGGAGGAAG	AAGTTCACTGGCTTGACATGGATG	100.6	0.999
RcCAMTA4	TGATGCCAACGAACCCACAAAG	GATTACGCCTTCCTGAATGATCTGAG	95.3	0.993
RcCAMTA5	TGTGCGTAGAGGTGAACCAGTAG	TGTGCGTAGAGGTGAACCAGTAG	96.5	0.997
RcACTIN	AGCGTGGCTATTCCTTCAC	GTACTTCTGGGCAACGGAA	94.6	0.984
